# Effect of Curcumol on the Fenestrae of Liver Sinusoidal Endothelial Cells Based on NF-*κ*B Signaling Pathway

**DOI:** 10.1155/2020/8590638

**Published:** 2020-05-28

**Authors:** Yang Zheng, Jiahui Wang, Jiaru Wang, Haiyuan Xie, Tiejian Zhao

**Affiliations:** ^1^Department of Medicine, Faculty of Chinese Medicine Science, Guangxi University of Chinese Medicine, Nanning, Guangxi 530021, China; ^2^College of Nursing, Guangdong Medical University, Dongguan, Guangdong 523000, China; ^3^Department of Physiology, College of Basic Medicine, Guangxi University of Chinese Medicine, Nanning, Guangxi 530021, China

## Abstract

**Objective:**

To study the effect of curcumol on liver sinusoidal endothelial cells (LSECs) and to analyze the mechanism of antihepatic fibrosis.

**Methods:**

The effects of drug intervention on cell proliferation rates were detected by MTT assay. The expression of NF-*κ*B was detected by RT-PCR and WB. The NF-*κ*B expression and entry into the nucleus were detected by immunofluorescence; scanning electron microscopy was used to observe the changes of LSECs fenestrae.

**Results:**

MTT results showed that the interference of cell proliferation in each group was small. RT-PCR showed that the expression of NF-*κ*B in the curcumol intervention group was significantly lower than that in the positive control group (*P* < 0.05). The WB detection found that, in the curcumol intervention group, the expression of pNF-*κ*B in the NF-*κ*B signaling pathway was significantly lower than that in the positive control group (*P* < 0.05). Scanning electron microscopy showed that the LSEC fenestrae were significantly improved compared with the positive control group.

**Conclusion:**

Curcumol may be one of the mechanisms of antihepatic fibrosis by inhibiting the activity of the NF-*κ*B signaling pathway and increasing the fenestrae of LSECs.

## 1. Introduction

Hepatic fibrosis is a common result of chronic liver disease and is mainly characterized by extensive deposition of extracellular matrix (ECM) [1]. Inflammation, chronic viral hepatitis, and liver damage are considered to be the leading causes of liver fibrosis. Liver injury leads to the loss of function of hepatic sinusoidal fenestrae, and hepatic sinusoidal thrombosis may promote liver fibrosis [2]. Liver sinusoidal endothelial cells (LSECs) play an essential role in the development of liver fibrosis [3]. LSECs are the primary boundary component of the hepatic sinusoidal wall, which are with fenestrae and the loose connection between cells, but without the basement membrane under endothelium. The structure of the hepatic sinusoidal wall is beneficial to regulate the material exchange between hepatocytes and hepatic sinusoidal blood. LSECs also have an active endocytic function and play an important role in regulating liver microcirculation and secreting extracellular matrix. In the early stage of liver fibrosis, LSECs show loss of fenestrae and subendothelial basement membrane formation. This phenomenon is named the capillarization of hepatic sinusoidal, which is an essential pathological change in the formation of liver fibrosis [4–6].

NF-*κ*B is a crucial inflammatory transcription factor that triggers the massive release of inflammatory cytokines, such as IL-6, TNF-*α*, and IL-8[7]. NF-*κ*B enhances the inflammatory response of the liver to form an “inflammatory waterfall” and eventually leads to the formation of liver fibrosis. Studies have found that hepatic stellate cell (HSC) collagen expression correlates with NF-*κ*B, inhibits NF-*κ*B expression and activity, and can significantly impede collagen expression [8]. Reportedly, NF-*κ*B has antiapoptotic effects, and some apoptosis stimuli activate NF-*κ*B. Activated NF-*κ*B blocks TNF-*α* mediated apoptosis [9]. As a fibrotic factor, NF-*κ*B enhances liver inflammation, participates in the regulation of proliferation and apoptosis of LSECs, and promotes collagen synthesis and secretion. The eNOS-NO-cGMP signaling pathway is an important pathway regulating the formation of fenestrae in LSECs [10, 11]. When the NF-*κ*B signaling pathway is activated, the I*κ*B kinase IKK is activated for phosphorylating and ubiquitinating the NF-*κ*B inhibitory protein I*κ*B*α*, resulting in a decrease in the cytoplasmic I*κ*B*α* content. Thus, the NF-*κ*B p65 subunit enters the nucleus from the inhibitory state to the activated state, activates the expression of multiple inflammatory factors, and regulates the transcription of the iNOS gene at the same time, leading to the disorder of the liver microcirculation [12–15].

In our previous study, we have found curcumol that suppresses HSC activation and contributes to the activation of HSC apoptosis during liver fibrosis [16]. Curcumol, a Chinese herbal medicine, promotes blood circulation for the removal of blood stasis. However, the effects of curcumol on LSECs remain undetermined.

We speculate that curcumol could reverse hepatic fibrosis by inhibiting the activity of the NF-*κ*B signaling pathway and increasing the fenestrae of LSECs. We aimed to elucidate that curcumol changes the structure of LSECs by inhibiting the activity of the NF-*κ*B signaling pathway, which might effectively reduce the pathogenesis of hepatic fibrosis.

## 2. Materials and Methods

### 2.1. Drugs and Reagents

Curcumol was purchased from Aladdin Chemical Company, MTT was purchased from Sigma Chemical Company, and carbon tetrachloride was purchased from Tianjin Fuyu Fine Chemical Company. Lipopolysaccharide was purchased from Sigma Chemical Company, PDTC from Selleck Chemical Company, Dimethyl Sulfoxide from Sigma Chemical Company, and DMEM and FBS from GIBCO Chemical Company.

### 2.2. Animals

The experiment was conducted with 8-week-old male Sprague Dawley (SD) rats from the Medical Laboratory of Guangxi Medical University. The animal certificate number was SCXK (Gui) 2016-0002, and the animal license number was SYXK (X) 2015-0001. The weight of the rats is 150–180 g. The animal research is carried out at the Animal Experimental Center of Guangxi University of Traditional Chinese Medicine and approved by the Animal Ethics Committee of Guangxi Traditional Chinese Medicine Institute (protocol number: 2016-12-02-1). All authors ensure that the guidelines and regulations for conducting all experiments were approved. All animals were tested under standard conditions (25°C and 12 hr light/dark cycles).

### 2.3. Cell Isolation and Culture

Rats were anesthetized by intraperitoneal injection with 3% pentobarbital sodium at a dose of 0.15 mL/100 g. The anesthetic was treated with an anticoagulant. Separation of LSECs was described by Oie et al. [17] using hepatic collagenase perfusion, differential centrifugation and Percoll density gradient sedimentation, and cell-selective adherence. The cells were identified by scanning electron microscopy and cultured in DMEM medium containing 10% FBS. Suitable conditions include moderate room temperature and 5% CO_2_ humidified air to maintain cells. After reaching 80∼90% confluence, cells were cultured on a 6-well plate with a density of 1 × 10^5^ cells per well for further experiments for 48 hr. To maintain the isolated LSEC phenotype, the culture was carried out, on the one hand, in DMEM containing 10% FBS and 10 ng/mL endothelial cell growth factor (ECGF). On the other hand, primary cells were seeded on collagen-coated coverslips for experiments and rapidly fixed by glutaraldehyde using an electron microscope.

### 2.4. Experimental Grouping and Intervention

The cells were divided into five groups. Group 1 cells were treated with 2% DMEM as the control group. Group 2 cells were treated with LPS (activate the NF-*κ*B signaling pathway) at a concentration of 5 *μ*g/mL for 3 hr as the positive group. Group 3 cells were treated with curcumol at a concentration of 45 *μ*g/mL for 48 hr as the curcumol group. Group 4 cells were pretreated with curcumol at a concentration of 45 *μ*g/mL for 48 hr and then treated with LPS 5 *μ*g/mL for 3 hr as the curcumol intervention group. Group 5 cells were pretreated with 25 *μ*g/mL PDTC (NF-*κ*B inhibitor) for 30 minutes and then treated with LPS at concentration 5 *μ*g/mL for 3 hr as the negative control group.

### 2.5. Cell Proliferation Experiment

The cell density of the isolated rat LSECs was adjusted to 5 × 10^4^ cells per mL with endothelial cell complete medium. Cells (100 *μ*L per well) in a 96-well plate (blank well was set at 37°C) were incubated overnight (100 *μ*L of sterile PBS was added to the wells around the wells). Each component was composed of three duplicate wells, cultured at 37°C, 5% CO_2_ saturated humidity for 48 hr. MTT (10 *μ*L) was added to each well and then incubated at 37°C for 4 hr. After the medium was aspirated, 150 *μ*L of DMSO was added for 10 min. The absorbance value OD568 of each well was determined by an enzyme labeling instrument. The cell proliferation rate of each group was calculated from the OD value.

### 2.6. Western Blots

The total protein was extracted and separated by SDS-PAGE and then transferred the PVDF membrane for incubation with the primary antibody at 4°C overnight. After being washed with PBS, the membranes were incubated with the corresponding HRP-labeled secondary antibody at 37°C for 2 hr and then were illuminated with ECL reagent. The gray value of the film was analyzed using BandScan.

### 2.7. Real-Time Quantitative PCR

The total RNA was extracted from LSECs using a total RNA extraction kit and then reverse-transcribed into cDNA using the Revastid First Strand cDNA Synthesis Kit. For qPCR, an SYBR Green or fluorescein qPCR master mix was used. The PCR primers were as follows: NF-*κ*B, forward, 5′-TGACGGGAGGGGAAGAAATC-3′, reverse, 5-TGAACAAACACGGAAGCTGG-3 (211 bp); GAPDH, forward, 5′-TCAAGAAGGTGGTGAAGCAGG-3′, reverse 5′-TCAAAGGTGGAGGAGTGGGT-3′ (115 bp). The reaction was carried out at 50°C for 2 minutes, 95°C, 10 min, 95°C for 30 sec, and 60°C for 30 sec. The PCR product was isolated using a 2% agarose for observation. GAPDH was used as an internal control. The ABI system was used for data analysis.

### 2.8. Immunofluorescence Observation

To investigate the expression and entry into nucleus of NF-*κ*B in LSECs, cells were washed 3 times with PBS for 3 minutes and then fixed with 4% paraformaldehyde solution for 15 min. The cells were then incubated with 0.5% Triton X-100 containing goat serum and incubated with fluorescently labeled goat anti-rabbit IgG antibody. An antifluorescence quenching seal and DAPI mounting media were mounted on glass slides and visualized using an fluorescent inverted microscope.

### 2.9. Scanning Electron Microscope Observation

To investigate the changes in fenestration in LSECs, we used a scanning electron microscope (SEM). All samples were subjected to scanning electron microscopy for 24 hr after administration. The preparation was washed twice with phosphate buffered saline (PBS) and then rapidly fixed with 2.5% glutaraldehyde for 1 hr. The formulation was washed twice with PBS, followed by 30%, 50%, 70%, and 80% ethanol, 4% C, and 90%, 95%, and 100% ethanol. The prepared samples were dehydrated for 10 minutes at room temperature. The formulation was then dried in 100% isoamyl acetate. Platinum (15 nm) was used as a coating for surface preparation samples. The sample was observed using a HITACHI scanning electron microscope (SU-8010).

### 2.10. Statistical Analysis

Data were analyzed using SPSS statistic 22.0 software and presented as the mean ± SD. ANOVA with Bonferroni posttests was used to evaluate significant differences between groups. *P* values < 0.05 were considered significant differences between independent groups.

## 3. Results

### 3.1. Isolation and Culture Results of LSECs

*Percoll Density Gradient Centrifugation*. The hepatocyte suspension is divided into density gradients of 4 different regions. The upper layer (between the top layer of the Percoll gradient and the 25% Percoll gradient layer) mainly contains debris, damaged cells, and a small amount of nonparenchymal cells. The boundary layer between 25% Percoll and 50% Percoll is rich in LSEC, with a small amount of unknown small white and red blood cells. The third layer (50% Percoll gradient zone) contains Kupffer cells (KC) and red blood cells. A large number of red blood cells were deposited in the bottom layer.

**Figure 1:**
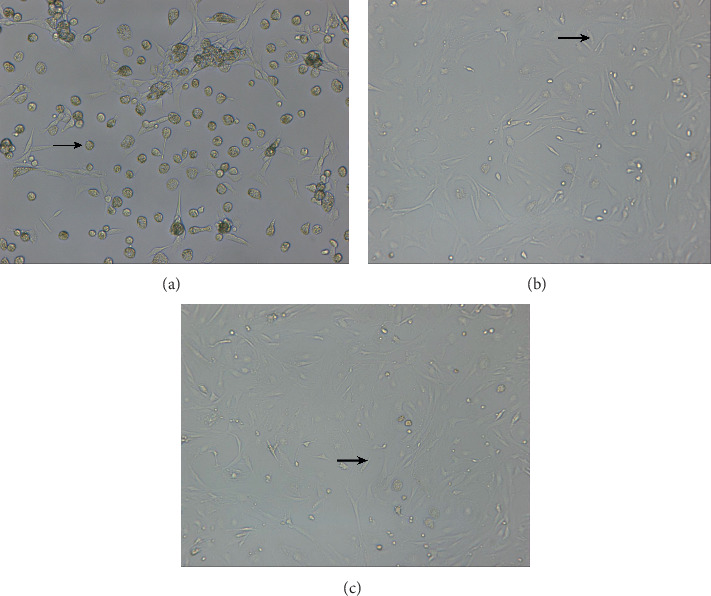
Results of liver sinusoidal endothelial cell culture. (a) Results of 12 hr isolated culture, inverted microscope X40. (b) Results of 36 hr isolated culture, inverted microscope X20. (c) Results of 60 hr isolated culture, inverted microscope X20.

After Percoll density gradient centrifugation, about 32 × 10^6^ cells of LSEC were obtained. After selective attachment, 22 × 10^6^ cells were obtained. As determined by trypan blue staining, the viability of the isolated LSEC cells reached 95–98%. The separate and cultured LSECs were observed under an optical microscope. At 12 hr, the cells were round and then gradually grew into an oval structure (as indicated by the arrow in Figure 1(a)). After 36 hr, the oval cells slowly gathered into a paving stone-like shape, and the cells gradually became a spinning cone (as indicated by the arrow in Figure 1(b)). After 60 hr, the cell morphology basically changed to a spinning cone (as indicated by the arrow in Figure 1(c)), and the cells grew better.

### 3.2. MTT Assay Showed That the Proliferation Rate of Each Group Was Higher

**Figure 2:**
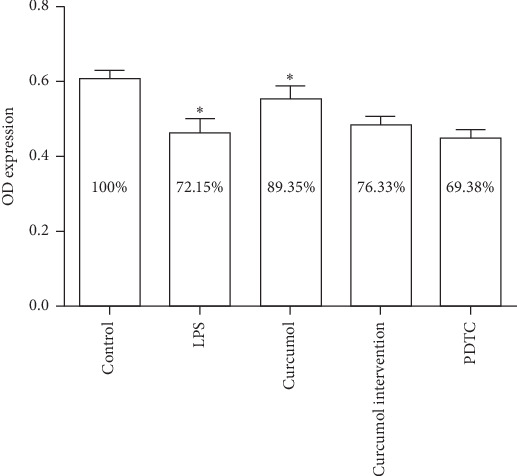
The expression of OD expression values in each group of cells. The data are analyzed using ANOVA with Bonferroni posttests through SPSS 22.0 software. The results are displayed in a bar graph as the mean ± SD. The percentage in the figure is the cell proliferation rate calculated based on the OD value. ^*∗*^*P* < 0.05 versus control.

The OD value of the curcumol group was statistically significant compared with the blank control group (*P* < 0.05). The OD value of the model group was statistically significant compared with the blank control group (*P* < 0.05). As shown in Figure 2. According to the cell proliferation rate formula (experimental group OD/blank control group OD), the proliferation rate of each group was greater than 60%, so the effect rate of each group of drugs on cell proliferation rate was relatively small, and the test results of subsequent experiments were reliable.

### 3.3. Curcumol Downregulated the Expression of NF-*κ*B mRNA and Phosphorylated NF-*κ*B

**Figure 3:**
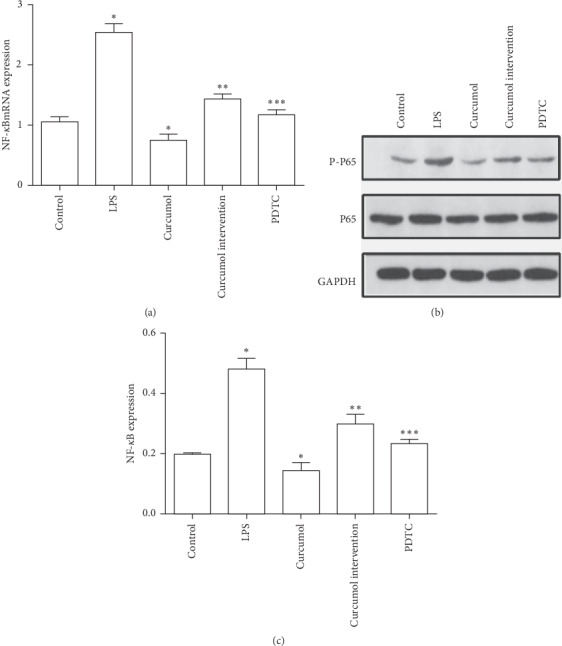
NF-*κ*B expression. (a) NF-*κ*B mRNA expression in each group. (b) Expression of phosphorylated NF-*κ*B in each group. (c) Expression of phosphorylated NF-*κ*B in each group. The data are analyzed using ANOVA with Bonferroni posttests through SPSS 22.0 software. The results are displayed in a bar graph as the mean ± SD. ^*∗*^*P* < 0.05 versus control, ^*∗∗*^*P* < 0.05 versus model group, ^*∗∗∗*^*P* < 0.05 versus curcumol intervention group.

The expression of NF-*κ*B mRNA in the curcumol intervention group was significantly lower than that in the model group (*P* < 0.05). The expression of NF-*κ*B mRNA in the model group was significantly higher than that in the blank control group (*P* < 0.05), and the expression of NF-*κ*B mRNA in the curcumol intervention group was significantly higher than that in the PDTC group (*P* < 0.05). The expression of NF-*κ*B mRNA in the curcumol group was significantly lower than that in the blank control group, and the difference was statistically significant (*P* < 0.05). RT-PCR detection showed that curcumol could inhibit the expression of NF-*κ*B Mrna, as shown in Figure 3.

The expression of phosphorylated NF-*κ*B in the curcumol intervention group was significantly lower than that in the model group, and the difference was statistically significant (*P* < 0.05), The expression of NF-*κ*B in the model group was significantly higher than that in the blank control group (*P* < 0.05). The expression of NF-*κ*B in the curcumol intervention group was significantly higher than that in the PDTC group (*P* < 0.05). The expression of NF-*κ*B in the curcumol group was significantly lower than that in the blank control group, and the difference was statistically significant (*P* < 0.05). WB assay showed that curcumol could inhibit the expression of phosphorylated NF-Κb, as shown in Figure 3. Curcumol inhibited the expression of NF-*κ*B mRNA and phosphorylated NF-*κ*B, which may be one of the mechanisms of its antifibrosis.

### 3.4. LPS Induces Defenestration of LSECs

**Figure 4:**
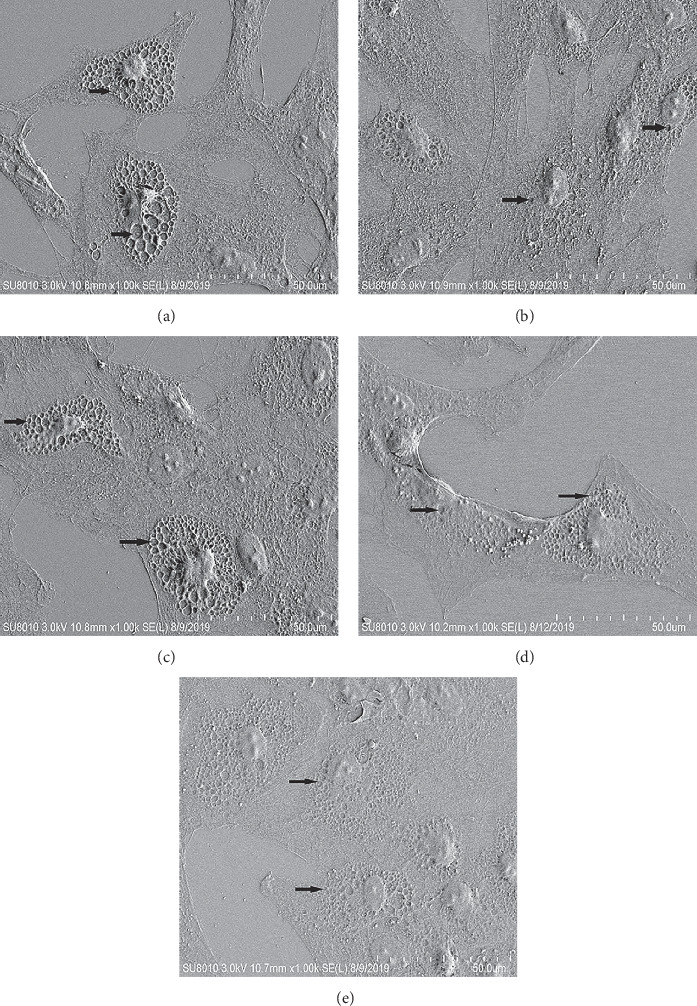
Curcumol returned fenestrae loss in in vitro study: the numbers of fenestrae were detected by scanning electron microscope examination. The arrow in the figure points to the fenestrae. The curcumol intervention group can significantly improve the opening of the model group fenestrae. Scale bar, 50 *μ*m. (a) Control. (b) LPS. (c) Curcumol. (d) Curcumol intervention. (e) PDTC.

In normal LSECs, there are a large number of open fenestrae. While the number of open fenestrae in the positive control group was reduced, the above results indicated that LPS mediates the change in the size and number of LSECs. Curcumol group has more open fenestrae than the control group, and the fenestrae size and number of cells in the curcumol intervention group increased compared with the positive control group, As shown in Figure 4. The results showed that curcumol could improve the opening of the fenestrae of the LSECs and improve the microcirculation in the liver.

### 3.5. Curcumol Can Reduce NF-*κ*B Translocation from the Cytosol to Nuclear Translocation 

**Figure 5:**
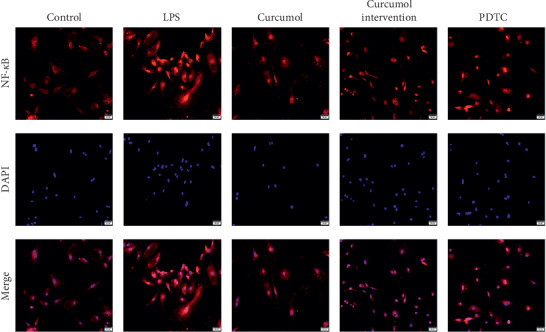
Curcumol decreases the levels of NF-*κ*B, as determined by immunofluorescence assay. LSECs were treated with curcumol after stimulation with LPS. Cells treated with vehicle were used as controls. NF-*κ*B (red) was widely observed in the cytoplasm of LSECs. The proteins were immunostained using cytokeratin-19 antibody and are shown in red. Nuclei were stained using DAPI (blue). Scale bar, 50 *μ*m.

To detect the expression of NF-*κ*B in LSECs and its translocation from cytosol to nuclear translocation, the immunofluorescence method was used to identify the expression of NF-*κ*B in LSECs. The expression of NF-*κ*B in curcumol group was lower than that in the blank control group, and that in curcumol intervention group was lower than that in the positive control group. Curcumol inhibits NF-*κ*B translocation from cytosol to nuclear translocation, as shown in Figure 5. The results showed that curcumol could inhibit the NF-*κ*B translocation from cytosol to nuclear translocation and regulate the expression of a series of downstream target genes, thereby improving the fenestrae of LSECs.

## 4. Discussion

Liver fibrosis is an essential stage for the further development of chronic hepatitis, and it is a pathophysiological process for the massive deposition of ECM [18]. It is well known that liver fibrosis eventually leads to cirrhosis and liver failure. Liver fibrosis is a common disease that requires effective treatment. Numerous studies have shown that liver fibrosis can be reversed [19]. Our previous studies have shown that curcumol has antifibrotic effects [16]. Our current data suggest that curcumol can restore the capillarization of hepatic sinusoidal in vitro.

Sinusoidal capillarization is mainly characterized by the formation of basement membranes and the loss of fenestrae openings of LSECs. LSECs constitute the blood vessel wall of the hepatic sinusoids and are the main population of nonparenchymal cells in the liver, accounting for approximately 50% of parenchymal cells. Under differentiated LSEC electron microscopy, its fenestrae structure can be seen, and there is no intact basement membrane under the fenestration endothelium. The fenestrae occupies 6% to 8% of the total surface area. This porous structure is found in liver parenchymal cells and blood substances. Exchange plays a key role. When sinusoidal capillarization occurs, the loss of LSECs leads to the reduced permeability of LSECs, and various metabolites do not easily enter the blood circulation, aggravating liver pathological damage. At the same time, liver cells and blood are weakened. The exchange of oxygen and nutrients eventually leads to hepatocyte damage and sinus collapse [20, 21]. LPS can induce liver sinusoidal endothelial cell activation. In contrast, curcumol reversed this pathology in LPS stimulated activated LSECs. In this study, we found that LPS promotes the expression of NF-*κ*B, while curcumol can downregulate the expression of NF-*κ*B. Combining the above data, we confirmed that curcumol reverses the pathological changes of LPS stimulated LSECs, such as sinusoidal capillarization, by downregulating the expression of NF-*κ*B.

NF-*κ*B is a pleiotropic transcription factor. NF-*κ*B in healthy cells is in an inactive state. It mainly binds to its inhibitor kappa B (I-*κ*B) as a p50/p65 dimer and exists in the cytoplasm. Various stimuli such as reactive oxygen species and cytokines can induce phosphorylation of I-*κ*B, dissociate it from p50/p65, activate NF-*κ*B, and transfer it into the nucleus. Combined with apoptosis-related genes to promote gene transcription and produce cells, apoptosis causes damage to the body. Oxidative stress is an important factor in inflammatory diseases of many diseases, and the NF-*κ*B inducible nitric oxide synthase (iNOS)-NO signaling pathway is an important oxidative stress pathway [22–24]. The activity of LSECs is also regulated by the NF-*κ*B/NO signaling pathway. LSECs can synthesize and secrete NO and ET, thereby regulating intrahepatic vasodilation and contraction, and play an important role in regulating hepatic sinus blood flow. NO has a powerful vasodilator effect, which directly stimulates soluble guanylate cyclase in a paracrine form, which increases cyclic guanylate, which causes Ca^2+^ reduction and vasodilation [25, 26]. Under physiological conditions, LSECs can express endothelial nitric oxide synthase (eNOS) and synthesize NO through eNOS, but the level of NO is low. Because of the strong ability of NO to diffuse and the long-term biological effects of metabolites, it is sufficient to maintain its normal blood perfusion. LSECs are extremely susceptible to damage by certain toxicants or drugs. Some studies have found that when LSECs is damaged, eNOS posttranslationally modifies abnormally, leading to a decrease in NO synthesis, while an increase in the expression of ET-1 with vasoconstriction can lead to HSC contraction [27]. HSC expresses the ET-1 receptor. ET-1 binds to the receptor to increase the Ca^2+^ content in the cell, causing contraction of myosin and the like. At the same time, it increases the expression of *α*-SMA and regulates HSC proliferation. Increased Ca^2+^ content and cell contraction induced by ET-1 are seen at any stage after HSC activation. ET-1 can also directly affect the hepatic sinusoids, cause liver microcirculation disorders, lead to liver cell damage, portal hypertension, and aggravate the liver disease. LSEC injury and dysfunction aggravate the liver injury and promote liver fibrosis and cirrhosis [28].

Sinusoidal capillarization precedes liver fibrosis. Sinusoidal capillarization leads to liver microcirculation disturbance. This lesion can activate hepatic stellate cells, leading to liver fibrosis [29]. Traditional Chinese medicine believes that blood stasis is the most important cause of liver fibrosis. Studies have shown that the capillarization may be the root cause of “blood stasis syndrome” in liver fibrosis [30]. Studies have found that [31] Curcuma is the top ten Chinese medicines commonly used in the treatment of chronic liver diseases. Curcumol is the main active ingredient of Guangxi's native medicinal herb Curcuma, and its antifibrotic effect is closely related to its pharmacological effect of promoting blood circulation and removing blood stasis. Our results show that the isolated and cultured LSECs grow better, have distinctive morphology, and grow regularly. The cells grow into a paving stone-like morphology and change from an initial round structure to an oval and spindle shape. The experiments provided the basis. MTT experiments showed that the cell proliferation in each group was excellent, indicating that various biological detection indicators were reliable. PCR, WB, and immunofluorescence analyses showed that the curcumol intervention group could inhibit the NF-*κ*B signaling pathway activated by LPS. Scanning electron microscopy results showed that curcumol could improve the fenestrae size and number of LSECs by inhibiting the activity of NF-*κ*B signaling pathway. Compared with the blank control group, the curcumol group can inhibit the activity of the NF-*κ*B signaling pathway, which indicates that when LSECs are at rest, curcumol can also inhibit the NF-*κ*B signaling pathway. Scanning electron microscopy observations show the hepatic sinusoids in the curcumol group. The number of LSECs fenestration was increased compared with the blank control group, which may be closely related to the pharmacological effect of curcumol on blood circulation and blood stasis. According to our research, curcumol can be used not only as an effective antifibrosis drug but also as a preventive agent for chronic liver diseases. Curcumol can inhibit the activity of NF-*κ*B signaling pathway of LSECs and improve the fenestrae of LSECs. It may be one of its molecular mechanisms for preventing and treating liver fibrosis. LSECs as targets for the prevention and treatment of chronic liver diseases will become an increasing concern.

## 5. Conclusion

Curcumol can regulate the structure of LSECs by inhibiting the activity of the NF-*κ*B signaling pathway in vitro.

## Data Availability

The data used to support the findings of this study are included within the article and are available from the corresponding author upon request.
